# Clinical benefits and risks of anticoagulation therapy according to the degree of chronic kidney disease in patients with atrial fibrillation

**DOI:** 10.1186/s12872-023-03236-5

**Published:** 2023-04-25

**Authors:** Min Soo Cho, Hyung Oh Choi, Ki Won Hwang, Jun Kim, Gi-Byoung Nam, Kee-Joon Choi

**Affiliations:** 1 Heart Institute, Asan Medical Center, University of Ulsan College of Medicine, Seoul, Republic of Korea; 2 Division of Cardiology, Department of Internal Medicine, Soonchunhyang University Hospital, Soonchunhyang University College of Medicine, 170, Jomaru-ro, Bucheon-si, Gyeonggi-do 14584 Republic of Korea; 3 Division of Cardiology, Department of Internal Medicine, Pusan National University Yangsan Hospital, Pusan National University of Medicine, Yangsan, Republic of Korea

**Keywords:** Atrial fibrillation, Anticoagulants, Renal insufficiency

## Abstract

**Background:**

The clinical benefits and risks of anticoagulation therapy in patients with chronic kidney disease (CKD) are still inconclusive. We describe the outcomes of patients with atrial fibrillation (AF) after anticoagulation therapy according to differences in creatinine clearance (CrCl). We also aimed to determine the patients who could benefit from anticoagulation therapy.

**Methods:**

This is a retrospective observational review of patients with AF who were managed at Asan Medical Center (Seoul, Korea) between January 1, 2006, and December 31, 2018. Patients were categorized into groups according to their baseline CrCl by Cockcroft–Gault equation and their outcomes were evaluated (CKD 1, ≥ 90 mL/min; CKD2, 60–89 mL/min; CKD3, 30–59 mL/min; CKD4, 15–29 mL/min; CKD 5, < 15 mL/min). The primary outcome was NACE (net adverse clinical events), defined as a composite of all-cause mortality, thromboembolic events, and major bleeding.

**Results:**

We identified 12,714 consecutive patients with AF (mean 64.6 ± 11.9 years, 65.3% male, mean CHA_2_DS_2_-VASc score 2.4 ± 1.6 points) between 2006 and 2017. In patients receiving anticoagulation therapy (n = 4447, 35.0%), warfarin (N = 3768, 84.7%) was used more frequently than NOACs (N = 673, 15.3%). There was a higher 3-year rate of NACE with renal function deterioration (14.8%, 18.6%, 30.3%, 44.0%, and 48.8% for CKD stages 1–5, respectively).The clinical benefit of anticoagulation therapy was most prominent in patients with CKD 1 (hazard ratio [HR] 0.49, 95% confidence interval [CI] 0.37–0.67), 2 (HR 0.64 CI 0.54–0.76), and 3 (HR 0.64 CI 0.54–0.76), but not in CKD 4 (HR 0.86, CI 0.57–1.28) and 5 (HR 0.81, CI 0.47–1.40). Among patients with CKD, the benefit of anticoagulation therapy was only evident in those with a high risk of embolism (CHA_2_DS_2_-VASc score ≥ 4, HR 0.25, CI 0.08–0.80).

**Conclusion:**

Advanced CKD is associated with a higher risk of NACE. The clinical benefit of anticoagulation therapy was reduced with the increasing CKD stage.

**Supplementary Information:**

The online version contains supplementary material available at 10.1186/s12872-023-03236-5.

## Background

The burden of atrial fibrillation (AF) increases with the global aging population, which is considered an important risk factor for embolic stroke [1, 2]. Renal dysfunction is significantly associated with incident AF and AF-associated complications, especially ischemic stroke [3]. Patients with coexisting AF and advanced chronic kidney disease (CKD) have a higher risk for ischemic stroke [4–7]. Oral anticoagulation therapy is the main strategy for thromboembolic prevention in patients with AF, but data on its efficacy and safety in patients with advanced CKD and those undergoing dialysis are sparse and contradictory [8]. In particular, the risk–benefit ratio between embolic protection and the risk of bleeding have not been established in patients with CKD. Specifically, the use of warfarin therapy in patients with end-stage renal disease (ESRD) has inherent limitations including a high risk of bleeding, hemorrhagic stroke, and vascular calcification; numerous interactions with other drugs; and the need for regular blood monitoring and dose adjustments, and non-vitamin K oral anticoagulants (NOAC) were not indicated in this group of patients [9, 10]. Therefore, there are many uncertainties regarding anticoagulation therapy in patients with CKD, and it is unclear whether it confers similar protection to reduce the risk of stroke in patients with AF who have advanced CKD or ESRD. In the current study, we describe the clinical outcomes of patients with AF after anticoagulation therapy according to CKD stage. We also aimed to determine patients with advanced CKD who could benefit from anticoagulation therapy.

## Methods

Data for the study subjects were extracted from the Asan BiomedicaL research Environment system, which is a clinical research data warehouse of the Asan Medical Center. In this system, all electronic medical records, international classification of disease codes, laboratory findings, imaging data, prescriptions, and follow-up data are available in anonymized form. Baseline and follow-up data of all patients were obtained from the system. This study conformed to the ethical guidelines of the Declaration of Helsinki. This study was approved by the Institutional Review Board of Asan Medical Center (2019 − 0215) on the 16 February 2019, which waived the need for informed consent owing to the retrospective nature of this study.

**Fig. 1:**
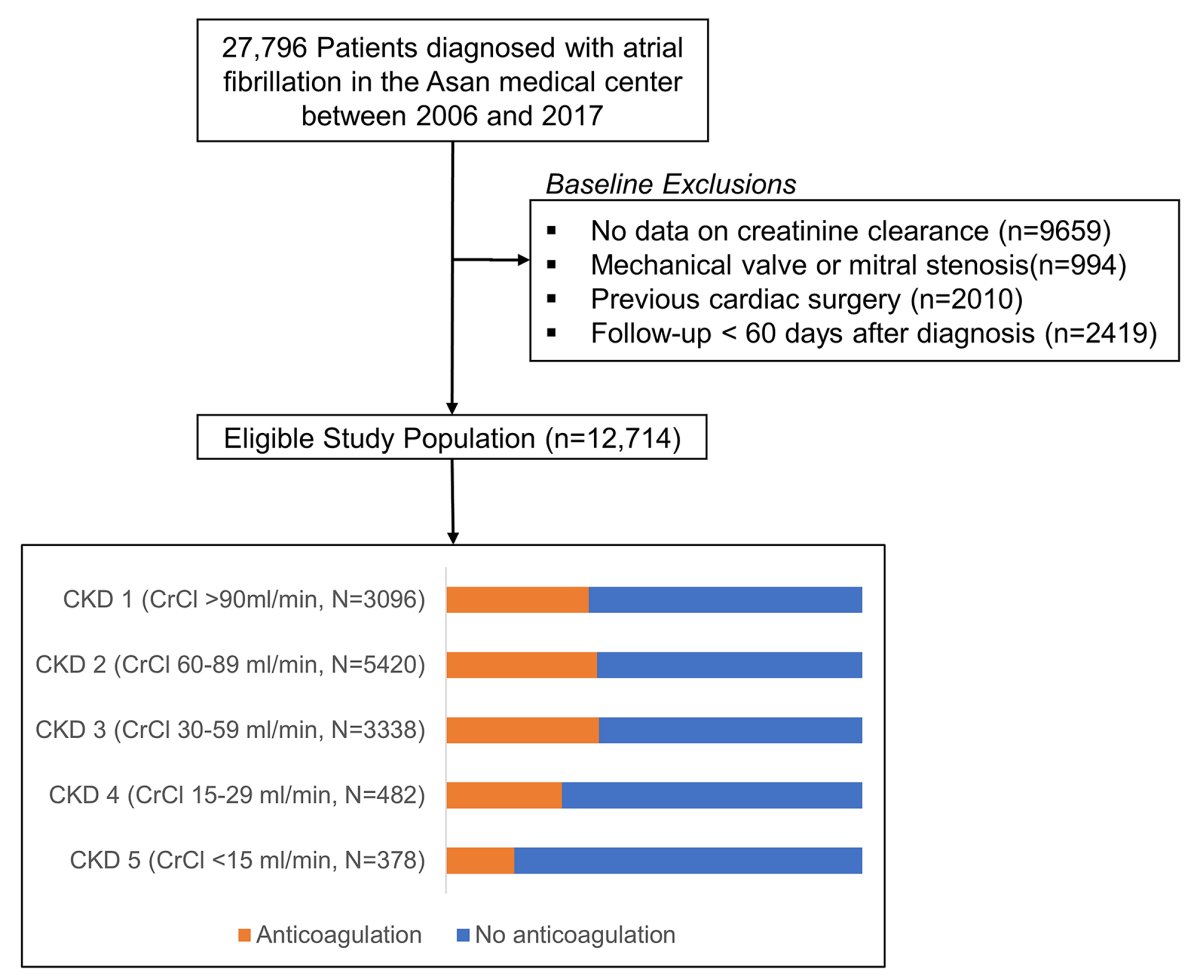
Flowchart of the study population

We identified 27,796 patients with a diagnosis of AF during the study period from January 1, 2006 to December 31, 2017. The following patients were excluded from the analysis: (1) no data on creatinine clearance; (2) with mechanical prosthetic valve or mitral stenosis; (3) with previous cardiac surgery; and (4) followed for less than 60 days (Fig. 1). The baseline renal function was calculated using the Cockcroft–Gault equation [11]. Patients were divided into five groups according to their creatinine clearance (CrCl) based on the Kidney Disease: Improving Global Outcomes (KDIGO) guidelines as follows: CKD 1, normal or increased CrCl (≥ 90 mL/min); CKD 2, mild reduction of CrCl (60–89 mL/min); CKD 3, moderate reduction of CrCl (30–59 mL/min); CKD 4, severe reduction of CrCl (15–29 mL/min); and CKD 5, kidney failure (< 15 mL/min).

In all patients, the risk of stroke was assessed according to the CHA_2_DS_2_-VASc score. Concomitant cardiovascular therapy was defined as the concomitant and continuous use of cardiovascular and anticoagulant medications for at least 30 days from study enrolment. Patients who received standard anticoagulation treatment (NOAC or warfarin) within 2 months of AF diagnosis were categorized into the anticoagulation therapy group. Patients were considered to be receiving standard anticoagulation treatment when they had a prescription record of at least 1 month for the continuous use of anticoagulants. Patients who did not undergo anticoagulation treatment were categorized into the control group. Patients were followed from the diagnosis of AF (non-anticoagulation group) or the start date of anticoagulation therapy (anticoagulation group).

The primary outcome was the net adverse clinical events (NACE), defined as a composite of death, ischemic stroke or systemic embolism, and major bleeding. The components of the primary outcome were analysed as secondary outcomes. Mortality was assessed by reviewing all available medical records and data from the National Health Insurance system. Ischemic stroke was diagnosed by an independent neurologist based on neurological symptoms and imaging evidence (computed tomography or magnetic resonance imaging). Systemic embolism was defined as a sudden loss of perfusion in a limb or organ, assessed using vascular imaging, ankle–brachial index, procedural findings, and laboratory findings along with clinical presentation. Major bleeding was defined when it was associated with a fatal outcome, it occurred in a critical area (intracranial, intraspinal, intraocular, retroperitoneal, intraarticular, pericardial, or compartment syndrome), it required a transfusion of ≥ 2 units of whole blood or packed red blood cells, or caused a drop in haemoglobin levels by ≥ 2 g/dL [12]. All study outcomes were adjudicated by independent research personnel blinded to the groups. The patients were censored as follows, whichever came first: (1) end of follow-up; (2) cross over of the treatment group (1.2% [52/4447] of patients with anticoagulation, 30.9% [2558/8267] without anticoagulation); and (3) occurrence of the primary outcome.

The baseline characteristics and clinical outcomes of the overall study population were evaluated. Descriptive statistics for categorical variables are presented as the absolute number and percentage, whereas those for continuous variables are presented as the mean ± standard deviation. Comparisons between groups were made using the chi-square test for categorical variables, and analysis of variance with post-hoc analysis with Tukey’s method or Kruskal-Wallis test as appropriate for continuous variables. Survival curves were estimated using the Kaplan–Meier method and were analysed using the log-rank test.

The Cox proportional hazards model was used to assess the relative risk of each variable on the study outcomes. We selected the variables for the multivariable model primarily based on their clinical relevance regarding the primary outcome; these variables included age, sex, body mass index, presence of hypertension, diabetes mellitus, stroke, intracranial haemorrhage, heart failure, vascular disease, malignancy, left atrial (LA) size, and left ventricular ejection fraction (LVEF). The proportional hazards assumption was tested by examining the log [-log survival] curves and partial (Schoenfeld) residuals. Subgroup analysis was performed in those patients with CKD 5 according to the CHA_2_DS_2_-VASc score, because this group of patients was not suitable for NOAC use and the results of anticoagulation were contradictory in previous studies. All statistical analyses were performed using R version 3.3.1 (R Institute for Statistical Computing, Vienna, Austria). A two-sided p-value of < 0.05 was considered statistically significant.

## Results

Between January 1, 2006 and December 31, 2017, 27,796 patients were diagnosed with AF. Of these, 15,082 patients who had missing data of CrCl, presence of valvular AF, and previous cardiac surgery or were followed up for ≤ 60 days after diagnosis were excluded (Fig. 1). A total of 12,714 patients were analysed and categorized into five groups according to the previously defined CrCl cutoff values (Table 1)—CKD 1 (≥ 90 mL/min), 24.5% (n = 3096); CKD 2 (60–89 mL/min), 42.8% (n = 5420); CKD 3 (30–59 mL/min), 26.4% (n = 3338); CKD 4 (15–29 mL/min), 3.8% (n = 482); and CKD 5 (< 15 mL/min), 2.9% (n = 378). Of the 378 CKD 5 patients, 300 (79.4%) underwent renal replacement therapy (haemodialysis, n = 279 [93.0%] and peritoneal dialysis, n = 21 [7.0%]). The median follow-up duration was 2.9 years (interquartile range, 1.3–4.9 years).

**Table 1:** Baseline characteristics of the study patients

CKD Grade	1	2	3	4	5	p
	(n = 3096)	(n = 5420)	(n = 3338)	(n = 482)	(n = 378)	
Age (years)	55 (47, 62)	65 (59, 71)	74 (69, 78)	77 (69, 83)	66 (58, 74)	< 0.001
Male	2366 (76.4)	3676 (67.8)	1820 (54.5)	233 (48.3)	212 (56.1)	< 0.001
Body mass index	25.8 (23.9, 28.1)	24.5 (22.7, 26.3)	23.2 (21.0, 25.2)	22.5 (20.2, 24.9)	22.8 (20.6, 25.1)	< 0.001
Hypertension	1796 (58.0)	3542 (65.4)	2568 (76.9)	407 (84.4)	351 (92.9)	< 0.001
Diabetes	438 (14.1)	869 (16.0)	778 (23.3)	214 (44.4)	210 (55.6)	< 0.001
Vascular disease	82 (2.6)	244 (4.5)	249 (7.5)	59 (12.2)	54 (14.3)	< 0.001
History of heart failure	279 (9.0)	575 (10.6)	640 (19.2)	154 (32.0)	106 (28.0)	< 0.001
Previous ischemic stroke	229 (7.4)	555 (10.2)	483 (14.5)	72 (14.9)	54 (14.3)	< 0.001
Previous ICH	36 (1.2)	75 (1.4)	59 (1.8)	11 (2.3)	12 (3.2)	0.009
CHA_2_DS_2_-VASc score	1 (0, 2)	2 (1, 3)	3 (2,4)	4 (3, 5)	3 (2, 5)	< 0.001
HAS-BLED score	1 (1, 2)	2 (1, 2)	2 (1, 3)	2 (2, 3)	3 (2, 4)	< 0.001
Paroxysmal AF	1811 (58.5)	2597 (47.9)	1345 (40.3)	207 (42.9)	223 (59.0)	< 0.001
History of malignancy	597 (19.3)	1421 (26.2)	970 (29.1)	124 (25.7)	85 (22.5)	< 0.001
LVEF	59 (54, 64)	59 (54, 63)	58 (50, 62)	57 (46, 62)	57 (46, 62)	< 0.001
LA A-P diameter	43 (38, 48)	44 (39, 49)	45 (39, 50)	44 (39, 50)	45 (41, 50)	< 0.001

Data are presented as the median (interquartile range) or number (%)

ICH: Intracranial haemorrhage, LVEF: Left ventricular ejection fraction, LVEDD: Left ventricular end-diastolic dimension, LA A-P: Left atrial anterior–posterior

The comparisons of the baseline characteristics according to CrCl values are shown in Table 1. Compared with patients in the lower CKD groups, those in the higher CKD groups were older, more likely to be female, and had a higher prevalence of baseline comorbidities including hypertension, diabetes, heart failure, and vascular disease. The CHA_2_DS_2_-VASc score tended to be higher in the higher CKD groups than in the lower CKD groups. The difference in baseline characteristics according to anticoagulation use is summarized in **Supplemental Table 1.** The patients using anticoagulation were characterized by older age, higher prevalence of comorbidities, and higher CHA_2_DS_2_-VASc or HAS-BLED scores. The prescription pattern of cardiovascular medications of each group is summarized in Table 2. Overall, the rate of antiarrhythmic drug use was higher in the lower CKD groups than in the higher CKD groups. The use of Ic drugs was dominant in the CKD 1 and 2 groups, whereas the use of amiodarone was dominant in the CKD 4 and 5 groups. Overall, the rate of anticoagulant use was higher in the lower CKD groups than in the higher CKD groups. The rate of anticoagulant use was significantly lower in the CKD 4 (27.8%) and CKD 5 (16.4%) groups than in the CKD 1–3 groups despite the high CHA_2_DS_2_-VASc scores in these groups (4.0 ± 1.5 in the CKD 4 group and 3.4 ± 1.7 in the CKD 5 group). Warfarin was prescribed more frequently in the CKD 4 and 5 groups than in the CKD 1–3 groups (91.0% and 96.8% in the CKD 4 and 5 groups, respectively).

**Table 2:** Drug prescription pattern according to chronic kidney disease grade

CKD Grade	1	2	3	4	5	p
	(n = 3096)	(n = 5420)	(n = 3338)	(n = 482)	(n = 378)	
Aspirin	1535 (49.6)	2565 (47.3)	1461 (43.8)	218 (45.2)	187 (49.5)	< 0.001
Clopidogrel	562 (18.2)	994 (18.3)	590 (17.7)	121 (25.1)	110 (29.1)	< 0.001
Beta blocker	1262 (40.8)	2182 (40.3)	1342 (40.2)	168 (34.9)	136 (36.0)	0.066
Calcium channel blocker	644 (20.8)	1233 (22.7)	731 (21.9)	101 (21.0)	81 (21.4)	0.314
Digoxin	456 (14.7)	1064 (19.6)	962 (28.8)	120 (24.9)	37 (9.8)	< 0.001
Amiodarone	603 (19.5)	989 (18.2)	707 (21.2)	139 (28.8)	116 (30.7)	< 0.001
Class Ic AAD^∫^	1317 (42.5)	1743 (32.2)	585 (17.5)	37 (7.7)	39 (10.3)	< 0.001
Anticoagulants	1061 (34.3)	1966 (36.3)	1224 (36.7)	134 (27.8)	62 (16.4)	
Warfarin	871 (82.1)	1668 (84.8)	1047 (85.5)	122 (91.0)	60 (96.8)	0.002
NOAC	190 (17.9)	298 (15.2)	177 (14.5)	12 (9.0)	2 (3.2)	
Dabigatran Rivaroxaban Apixaban Edoxaban	107 (56.3) 51 (26.8) 31(16.3) 1 (0.5)	125 (41.9) 109 (36.6) 64 (21.5) 0 (0.0)	58 (32.8) 86 (48.6) 32 (18.1) 1 (0.6)	3 (25.0) 4 (33.3) 5 (41.7) 0 (0.0)	0 (0.0) 0 (0.0) 2 (100.0) 0 (0.0)	< 0.001

Data are presented as a number (%)

CKD: Chronic kidney disease, AAD: Antiarrhythmic drug, NOAC: Non-vitamin K oral anticoagulants

**Fig. 2:**
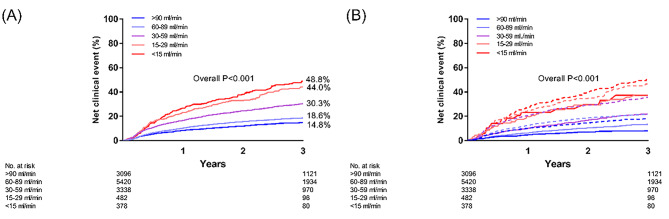
Kaplan–Meier plots of the rate of net adverse clinical events (NACE) according to creatinine clearance in all cohorts. (A) Rate of NACE according to the stage of chronic kidney disease. (B) Rate of NACE in patients treated with (bold line) or without (dotted line) anticoagulation therapy according to the stage of chronic kidney disease

**Fig. 3:**
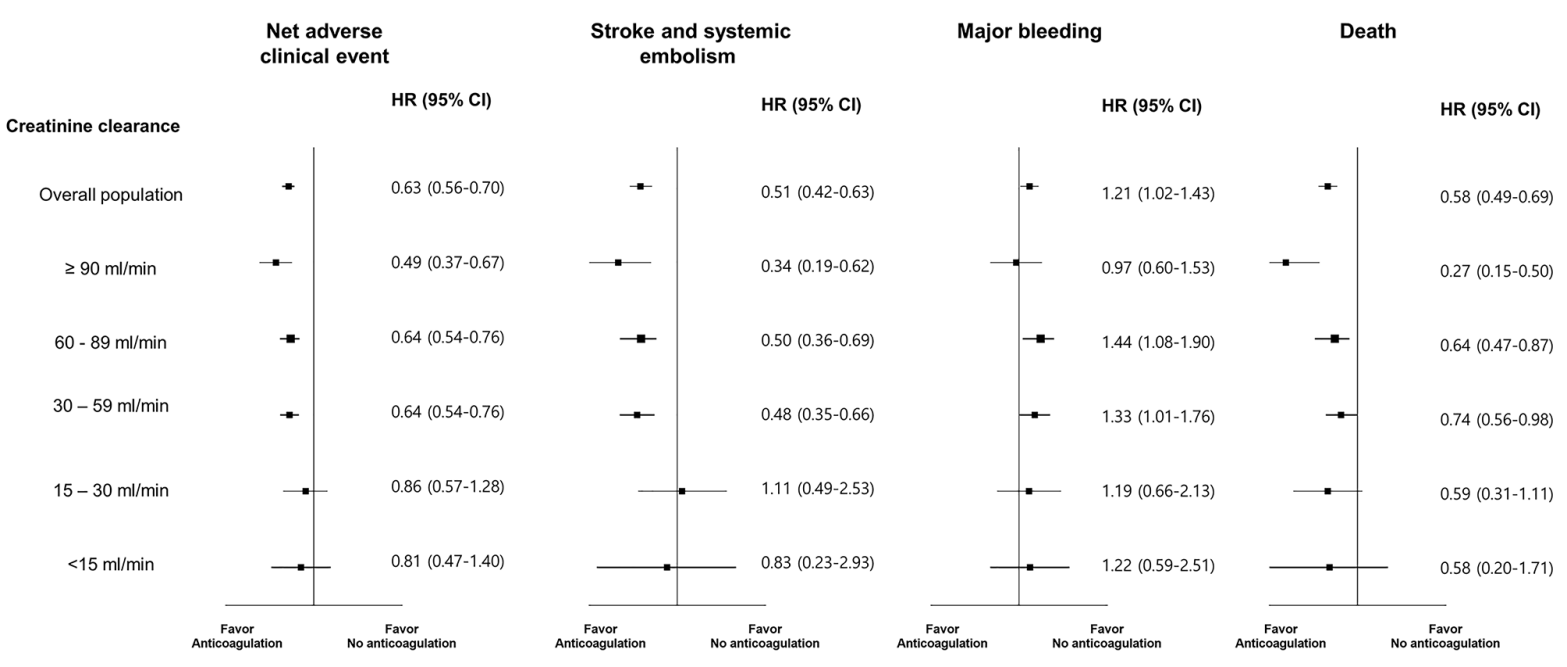
Forest plot demonstrating the risk of net clinical adverse events (stroke or systemic embolism, bleeding, and death), by anticoagulation therapy according to the stage of chronic kidney disease

There was a trend for a progressive increase in the incidence of NACE as the renal function deteriorated (Fig. 2). In particular, the CKD 5 group had the highest rate of net clinical outcomes (**Supplemental Table 2**). There was a trend for a lower incidence of NACE in the anticoagulation group than in the control group in all the renal function strata (Fig. 2). The univariable and multivariable Cox proportional model was established by incorporating all clinically relevant variables (**Supplemental Table 3**). The multivariable adjusted hazard ratios (HRs) of NACE are shown in Fig. 3. In the groups with a CrCl ≥ 30 mL/min, anticoagulation therapy was associated with fewer NACE. However, the benefit of anticoagulation therapy became insignificant in the CKD 4 and 5 groups. Regarding the secondary outcomes, the benefit of anticoagulation therapy in terms of protection from thromboembolic events or mortality was maintained in the CKD 1–3 groups; however, it became insignificant in the CKD 4–5 groups. Furthermore, there was no significant difference in major bleeding between the CKD groups.

**Fig. 4:**
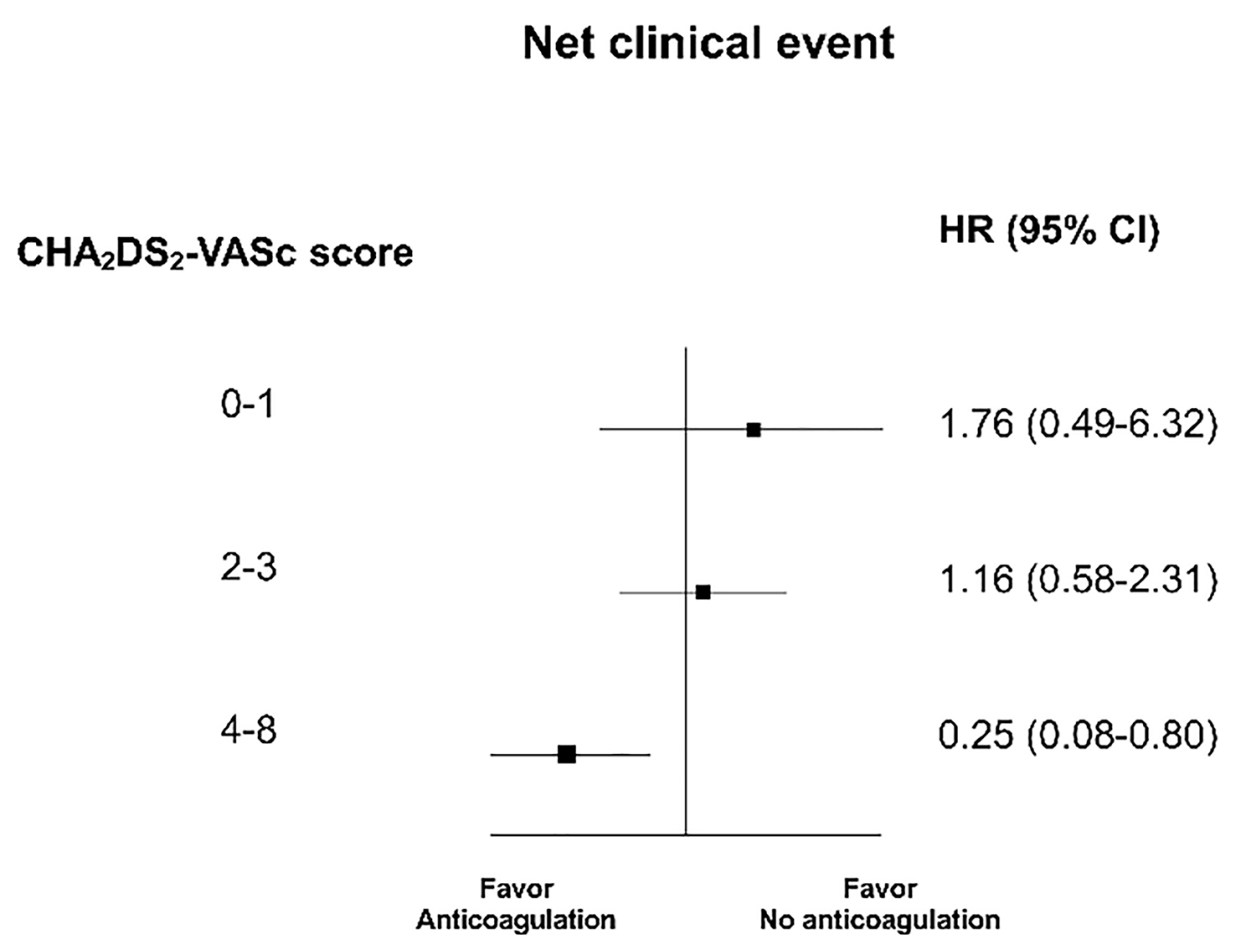
Risk of the net adverse clinical event death by anticoagulation therapy according to the CHA_2_DS_2_-VASc score in patients with chronic kidney disease stage 5

In the CKD 5 group, patients with a CHA_2_DS_2_-VASc score of ≥ 4 had significantly more frequent composite clinical events than those with a CHA_2_DS_2_-VASc score of < 4 (**Supplemental Fig. 1**). Furthermore, in very high-risk patients with a CHA_2_DS_2_-VASc score of 4–8, anticoagulation therapy was associated with a lower risk of NACE (HR 0.25, 95% CI 0.08–0.80, p = 0.027, Fig. 4).

## Discussion

The major findings of the current study were as follows: (1) a significant number of patients with AF also had CKD; (2) patients with advanced CKD had more comorbidities and a higher risk for NACE; (3) the benefit of anticoagulation therapy was most prominent only in patients with normal kidney function to moderate CKD (CKD 1–3); and (4) patients with ESRD (CKD 5) and those with the highest embolic risk (CHA_2_DS_2_-VASc score ≥ 4) may benefit from anticoagulation therapy.

AF and CKD share common cardiovascular risk factors including hypertension and diabetes, and have a close bidirectional relationship; kidney dysfunction is predisposed to the incidence of AF, whereas AF is associated with an increased incidence of renal dysfunction and an increased risk of CKD development and progression [13, 14]. The prevalence of AF among patients with CKD who do not yet require dialysis is generally two- to three-fold greater than that in the general population [11, 15, 16]. The relationship between AF and CKD can be explained by several factors. First, individuals with reduced kidney function are more likely to develop poor control of blood pressure, which leads to left ventricular hypertrophy, poor ventricular compliance, and atrial stretch and fibrosis [16, 17]. Second, both conditions were associated with endothelial injury or dysfunction [17]. Third, the pathological activation of the intrarenal renin–angiotensin–aldosterone system leads to atrial fibrosis and electrical remodelling, partly mediated through the increased secretion of transforming growth factor-β1 [16, 18]. Lastly, systemic inflammation and sympathetic overactivity are known to affect the genesis of both conditions [17, 19].

CKD in patients with AF is associated with a substantial increase in the incidence of thromboembolism as well as an increased risk of bleeding events. CKD and AF are associated with a prothrombotic state due to alterations in all components of Virchow’s triad— abnormal blood flow, abnormal blood constituents, and abnormal vessel walls [17]. Thus, patients with coexisting AF and CKD have a higher risk for stroke, thromboembolism, and mortality than those with CKD or AF alone [10, 20]. In a previous study, the risk of stroke gradually increased with progressively lower CrCl values at baseline, and the highest risk was observed in patients with AF and ESRD [21]. In contrast, Iseki et al. reported an inverse relationship between renal function and that the risk of cerebral haemorrhage in patients with CKD undergoing dialysis was 10-fold higher [22]. Furthermore, a pro-haemorrhagic state for renal failure was associated with disorders of the coagulation cascade, activation of fibrinolysis, reduced platelet function, and alterations in platelet–vessel wall interactions [17, 23]. In summary, anticoagulation in AF patients with advanced CKD was a high risk factor for thrombosis and bleeding, and thus is a clinical dilemma.

The current AF guidelines have adopted the use of the CHA_2_DS_2_-VASc score to identify patients at low risk for stroke [2]. However, the CHA_2_DS_2_-VASc score was developed using a general population and does not consider renal function as an important variable. Data on the use of stroke risk assessment tools for patients with advanced CKD or those undergoing dialysis are limited [24, 25]. Furthermore, it is unclear whether anticoagulation therapy can improve clinical outcomes in patients with advanced CKD or those undergoing dialysis. Some studies have reported a higher risk for bleeding without any benefit in terms of reducing the risk of ischemic stroke [26–30]. A meta-analysis including CHA_2_DS_2_-VASc scores or recent Korean data also demonstrated unfavourable outcomes in patients undergoing dialysis following anticoagulation treatment [9, 31].

In the current study, NACE was the primary outcome, encompassing stroke, bleeding, and death. By combining these clinical events, NACE offers a more comprehensive evaluation of the risk–benefit profile of anticoagulation therapy and is therefore used in major clinical trials, particularly those seeking a balance between efficacy and bleeding risk [32, 33]. We developed a multivariable model that incorporates known risk factors (components of the CHA_2_DS_2_-VASc score) and new risk factors (malignancy, left atrial size, and left ventricular ejection fraction) that have been evaluated in previous studies [34, 35]. By employing suitable primary outcomes and multivariable modelling, our study offers insightful guidance on the application of anticoagulation therapy for patients with advanced CKD.

Importantly, although the incidence of NACE was increased with advanced stage of CKD, clinical benefit after anticoagulation therapy disappeared in patients with CKD 5. Previous retrospective studies have suggested the clinical benefit of oral anticoagulation in patients with mild-to-moderate CKD [20]. However, in CKD 5 patients on renal replace therapy, oral anticoagulation was not associated with net clinical benefit, because the benefits of stroke prevention or death were counterbalanced by excessive rates of bleeding [10]. Therefore, there is a lack of high-quality evidence-based recommendations and routine anticoagulation therapy is not recommended for this population [2]. Although NOAC is expected to be a safe alternative to warfarin,[36]. the recently published randomized RENAL-AF trial demonstrated no clinical advantage of apixaban over warfarin [37]. Especially, the high annual incidence (30%/year) of clinically relevant bleeding in both arms suggested the net clinical benefit of anticoagulation in CKD 5 should be reconsidered and more selective approaches are required. In this regard, our analysis provides some valuable guidance on managing CKD 5 patients. Based on our data, only CKD 5 patients with the highest thromboembolic risk (CHA_2_DS_2_-VASc scores ≥ 4) are expected to benefit from anticoagulation therapy, in terms of NACE. We believe that our data clearly identified a specific group of CKD 5 patients who are indicated for anticoagulation. Further studies are needed to define anticoagulation suitable CKD 5 patients more precisely.

This study had several limitations. First, inherent selection bias was unavoidable due to the retrospective nature of the study. Second, the incidence of clinical outcomes may have been underestimated because the analysis was fundamentally based on in-hospital data. Third, the findings of the current study should be generalized with caution as the number of patients with moderate-to-severe CKD was limited, and all patients were followed up in a tertiary hospital. Fourth, as most of the patients were enrolled before NOAC reimbursement in South Korea, overall outcomes might be different from the current era of wide NOAC use. Fifth, the rate of anticoagulation in the entire Korean population was quite low (< 40%) before the introduction of NOACs [38]. In addition, indications for anticoagulation were not consistent throughout the study period as the patients were accrued from 2006 onwards. Finally, although cross-over occurred in 20.5% of the study population, we cannot assess the exact reason for this because of the retrospective nature of the study. Despite these limitations, our results are meaningful and show the effect of anticoagulant treatment in very high-risk patients with advanced CKD or those undergoing dialysis.

## Conclusion

In our study, the rate of NACE increased in line with an increase in the severity of CKD. The benefits of anticoagulation therapy were evident in patients with normal renal function to moderate CKD (CKD 1–3), but not in overall patients with advanced CKD (CKD 4–5). For patients with end-stage renal disease (CKD 5), anticoagulation therapy might be beneficial only in patients at very high-risk for embolism (CHA_2_DS_2_-VASc score ≥ 4).

## Electronic supplementary material

Below is the link to the electronic supplementary material.

Supplementary tables

## Data Availability

The original contributions presented in the study are included in the article/Supplementary Material and further inquiries can be directed to the corresponding author. The detailed data related to the findings of this study are available from the corresponding author upon reasonable request.
