# Supplementary material for: Clinical benefits and risks of anticoagulation therapy according to the degree of chronic kidney disease in patients with atrial fibrillation
Source: BMC Cardiovasc Disord. 2023 Apr 25;23:209. doi: 10.1186/s12872-023-03236-5 (PMC10131393; doi:10.1186/s12872-023-03236-5)

Supplemental table 1. Baseline characteristics according to the anticoagulation use

| Variables | With  anticoagulation | Without  Anticoagulation | P value |
| --- | --- | --- | --- |
|  | (n = 4447) | (n = 8267) |  |
| Age (years) | 64.2 ± 12.0 | 64.9 ± 11.9 | 0.003 |
| Male | 2867 (64.5) | 5440 (65.8) | 0.137 |
| Body mass index | 24.8 ± 3.4 | 24.4 ± 6.4 | <0.001 |
| Hypertension | 3292 (74.0) | 5372 (65.0) | <0.001 |
| Diabetes | 809 (18.2) | 1700 (20.6) | 0.001 |
| Vascular disease | 203 (4.6) | 485 (5.9) | 0.002 |
| History of heart failure | 823 (18.5) | 931 (11.3) | <0.001 |
| Previous ischemic stroke | 690 (15.5) | 703 (8.5) | <0.001 |
| Previous ICH | 40 (0.9) | 153 (1.9) | <0.001 |
| CHA_2_DS_2_-VASc score | 2.6 ± 1.7 | 2.3 ± 1.6 | <0.001 |
| HAS-BLED score | 1.7 ± 1.1 | 1.8 ± 1.0 | 0.023 |
| Paroxysmal AF | 2166 (48.7) | 4017 (48.6) | 0.915 |
| History of malignancy | 723 (16.3) | 2474 (29.9) | <0.001 |
| LVEF | 53.7 ± 12.9 | 57.5 ± 9.7 | <0.001 |
| LA A-P diameter | 46.1 ± 8.2 | 43.3 ± 8.2 | <0.001 |

Data are presented as mean ± standard deviation or number (%)

Supplemental table 2. Clinical outcomes according to renal function.

|  | With anticoagulation | | Without anticoagulation | |  |
| --- | --- | --- | --- | --- | --- |
|  | Crude event  no. (%) | Event rate  (/100 person-years) | Crude event  no. (%) | Event rate  (/100 person-years) | P-value |
| CKD1 (N=3096)  Stroke or systemic embolism  Major bleeding  Death  Net clinical adverse event | N=1061  16  35  13  63 | 0.5  0.9  0.4  1.6 | N=2035  74  77  178  297 | 0.7  0.7  1.7  2.8 | 0.001  0.493  <0.001  <0.001 |
| CKD2 (N=5420)  Stroke or systemic embolism  Major bleeding  Death  Net clinical adverse event | N=1966  98  165  88  291 | 0.9  1.4  0.8  2.6 | N=3454  202  151  346  627 | 1.1  0.8  1.9  3.6 | 0.181  <0.001  <0.001  0.002 |
| CKD 3 (N=3338)  Stroke or systemic embolism  Major bleeding  Death  Net clinical adverse event | N=1224  83  158  129  302 | 1.4  2.6  2.1  5.0 | N=2114  202  154  308  575 | 2.4  1.8  3.6  6.8 | 0.006  <0.001  0.001  0.110 |
| CKD 4 (N=482)  Stroke or systemic embolism  Major bleeding  Death  Net clinical adverse event | N=134  14  27  19  48 | 2.7  5.2  3.7  9.3 | N=348  27  50  73  120 | 2.5  4.6  6.7  11.1 | 0.343  0.121  0.089  0.782 |
| CKD 5 (N=378)  Stroke or systemic embolism  Major bleeding  Death  Net clinical adverse event | N=62  6  14  10  28 | 2.3  5.3  3.8  8.8 | N=316  33  62  59  137 | 3.2  5.1  6.8  13.5 | 0.856  0.595  0.636  0.793 |

Supplemental Table 3. Predictors of net adverse clinical events in univariable and multivariable analysis.

|  | Univariable analysis | | Multivariable analysis | |
| --- | --- | --- | --- | --- |
| Variables | HR (95% CI) | *P* Value | HR (95% CI) | *P* Value |
| Anticoagulation | 0.56 (0.51-0.62) | <0.001 | 0.62 (0.56-0.69) | <0.001 |
| Age (vs <65 years) |  |  |  |  |
| ≥75 years | 2.32 (2.07-2.50) | <0.001 | 1.25 (1.13-1.39) | <0.001 |
| ≥65 and <75 years | 1.70 (1.53-1.88) | <0.001 | 1.53 (1.36-1.72) | <0.001 |
| Female | 1.00 (0.91-1.10) | 0.977 | 1.14 (1.03-1.25) | 0.006 |
| Body mass index | 0.90 (0.89-0.91) | <0.001 | 0.92 (0.91-0.94) | <0.001 |
| Hypertension | 1.16 (1.06-1.28) | 0.002 | 1.03 (0.93-1.14) | 0.582 |
| Diabetes mellitus | 1.91 (1.74-2.10) | <0.001 | 1.52 (1.38-1.68) | <0.001 |
| Previous ischemic stroke | 1.49 (1.32-1.69) | <0.001 | 1.52 (1.33-1.73) | <0.001 |
| Previous ICH | 2.64 (2.09-3.35) | <0.001 | 1.84 (1.44-2.34) | <0.001 |
| History of heart failure | 1.45 (1.29-1.62) | <0.001 | 1.35 (1.18-1.54) | <0.001 |
| Vascular disease | 1.69 (1.45-1.97) | <0.001 | 1.26 (1.07-1.48) | 0.004 |
| History of malignancy | 4.13 (3.78-4.51) | <0.001 | 3.74 (3.42-4.10) | <0.001 |
| LA size, per 5mm | 1.01 (0.98-1.03) | 0.580 | 1.05 (1.02-1.08) | <0.001 |
| LVEF, per 5% | 0.97 (0.95-0.98) | <0.001 | 0.96 (0.94-0.99) | 0.004 |

ICH, intracranial hemorrhage; LA, left atrial; LVEF, left ventricular ejection fraction

Supplemental figure 1. Kaplan-Meier plots for rate of net adverse clinical events (NACE) according to CHA_2_DS_2_-VASc score in patient with chronic kidney disease stage 5. (A) Rate of NACE according to the CHA_2_DS_2_-VASc score. (B) Rate of NACE in patients with (bold line) or without (dotted line) anticoagulation according to the CHA_2_DS_2_-VASc score.


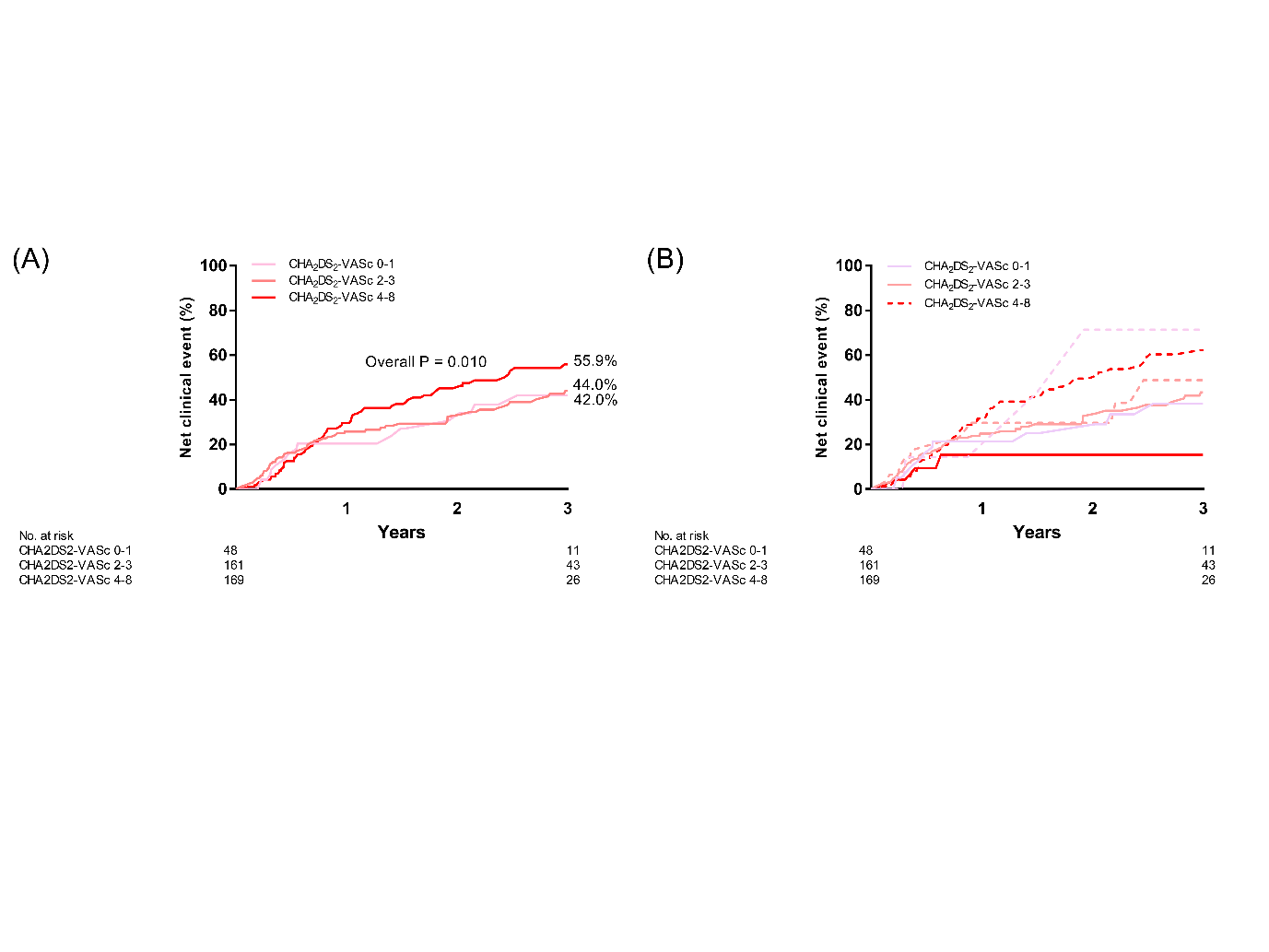

Supplement: Supplementary file 1 — Supplementary tables [file 12872_2023_3236_MOESM1_ESM.docx]
